# Trends in the prevalence of kidney stones among U.S. adults with obesity from 2007 to 2020

**DOI:** 10.1097/JS9.0000000000002693

**Published:** 2025-06-20

**Authors:** Wenshuang Li, Junlong Huang, Zheng Liu, Ruixiang Luo, Ziqiao Wang, Chi Zhang, Bolong Liu, Xiangfu Zhou

**Affiliations:** Department of Urology, Third Affiliated Hospital of Sun Yat-Sen University, Guangzhou, P. R. China

**Keywords:** kidney stones, NHANES, obesity, prevalence, trend

## Abstract

**Background:**

Kidney stones are a significant health concern in the United States, and their increasing prevalence is linked to increasing obesity rates. This study aimed to assess the trends in kidney stone prevalence among U.S. adults with obesity from 2007 to 2020 using National Health and Nutrition Examination Survey (NHANES) data.

**Materials and methods:**

This cross-sectional analysis used anonymized NHANES data from six cycles (2007–2020). Prevalences were estimated using NHANES sample weights; age-standardized prevalences were determined using 2020 census data. Survey-weighted multivariate logistic regression and linear regression models were used to assess risk factors and trends, respectively. Subgroup analyses were performed according to sex, race/ethnicity, and poverty-income ratio (PIR).

**Results:**

The overall age-standardized prevalence of kidney stones increased from 9.4% (2007–2008) to 10.2% (2017–2020). The prevalence among individuals with obesity significantly increased from 11.0% to 12.5% (*P* for trend = 0.035). The prevalence of kidney stones in females with obesity significantly increased from 8.8% to 11.5% (*P* for trend = 0.042), whereas males with obesity showed a slight increase (13.4% to 14.0%). Racial/ethnic disparities were evident among those with obesity: non-Hispanic Whites showed a modest increase (12.4% to 14.2%), Hispanics exhibited a notable increase (7.5% to 10.9%; *P* for trend = 0.017), and non-Hispanic Blacks had a stable prevalence that increased slightly (5.9% to 6.8%; *P* for trend = 0.304). The prevalence increased (10.2% to 12.9%; *P* for trend = 0.051) among individuals with obesity and high PIRs and decreased (12.8% to 11.4%) among those with low PIRs.

**Conclusions:**

This study highlights an upward trend in the prevalence of kidney stones among U.S. adults with obesity, from 2007 to 2020. Our findings emphasize the need for targeted public health strategies to address this issue, especially among populations at higher risk due to obesity and socioeconomic factors.

## Introduction

Kidney stones represent a significant urological issue in the United States, affecting approximately one in ten individuals^[1]^. This condition imposes considerable healthcare costs and adversely affects patients’ quality of life^[2]^. Alarmingly, recent studies have reported an increased prevalence of kidney stones^[3,4]^. Meanwhile, the prevalence of obesity – a well-recognized risk factor for kidney stone formation – has risen sharply in recent decades^[5,6]^. Obesity contributes to various metabolic disturbances linked to kidney stones, including altered calcium metabolism, elevated uric acid levels, and an increased diabetes risk^[7,8]^. Despite the established association between obesity and kidney stones, temporal trends in the prevalence of kidney stones specifically within obese populations remain inadequately characterized.

**HIGHLIGHTS**

- From 2007 to 2020, the prevalence of kidney stones significantly increased among U.S. adults, particularly among females.
- The kidney stone prevalence among individuals with obesity significantly increased from 11.0% to 12.5%.
- Significant demographic disparities in the prevalence of kidney stones were identified among adults with obesity, with notable variations according to sex, race/ethnicity, and socioeconomic status.

The National Health and Nutrition Examination Survey (NHANES) offers a valuable resource for investigating this issue, given its rigorous sampling methods and extensive longitudinal data representative of the noninstitutionalized civilian population in the United States^[9]^. Previous NHANES analyses documented a troubling increase in the prevalence of kidney stones from 5.2% in 1988–1994 to 8.8% in 2007–2010^[1,10]^. Concurrently, the prevalence of obesity has markedly increased as well, with rising trends from 2005 to 2014^[5]^. However, most studies have focused on overall population trends of the prevalence of kidney stones, and the complex interactions between obesity-related pathophysiology and changes in the prevalence of kidney stones over time have been overlooked.

This study aimed to examine the trends in the prevalence of kidney stones among adults with obesity in the United States from 2007 to 2020, exploring potential contributing factors and public health implications. Understanding these trends is crucial for developing effective prevention strategies and managing this growing health concern in an increasingly obese population.

## Materials and methods

### Study population

This study utilized data from the NHANES to assess the prevalence and trends of kidney stones. Publicly available, de-identified NHANES data were used; therefore, no additional institutional review board (IRB) approval was required, in accordance with the NIH guidelines. This study was reported in accordance with the STROCSS criteria^[11]^.

**Figure 1.**
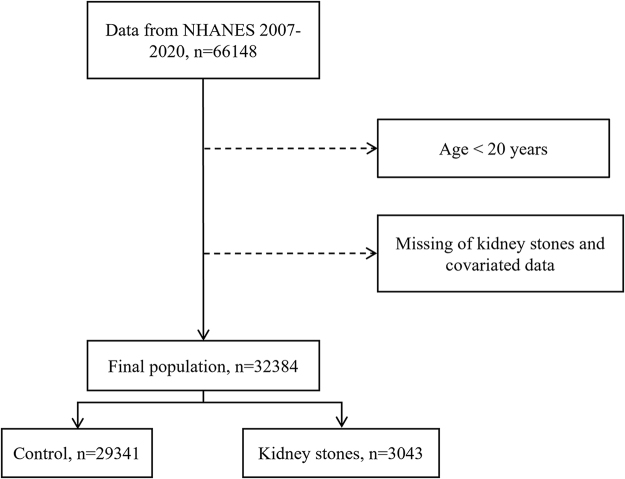
Flowchart of the participant enrollment from the NHANES 2007–2020.

We conducted a cross-sectional analysis of six NHANES cycles, from 2007 to 2020. During this period, 66,148 individuals participated in the NHANES. The exclusion criteria were an age <20 years and missing data on the history of kidney stones or relevant covariates. A total of 32,384 participants were included in the study, comprising 29,341 individuals in the control group and 3,043 individuals with a history of kidney stones (Fig. 1).

### Assessment of kidney stones and covariates

The primary outcome was the self-reported prevalence of kidney stones. Participants were asked, “Has a doctor or other health professional ever told you that you have kidney stones?” Those who answered “yes” had a history of kidney stones.

The covariates included age (20–39, 40–59, and ≥60 years), sex (male/female), and race/ethnicity. Following the analytical guidelines, the “Mexican American” and “Hispanic” categories were combined. Therefore, race/ethnicity was classified into four categories: non-Hispanic White, non-Hispanic Black, Hispanic, and Other. The poverty-income ratio (PIR) was categorized into two groups: PIR ≥2 and PIR <2. The body mass index (BMI) was categorized as nonobese (<30 kg/m^2^) or obese (≥30 kg/m^2^). Additional covariates included the educational level (greater than high school vs. less than high school), marital status (living alone vs. married/living with a partner), alcohol consumption (yes/no), smoking status (never, former, or current), physical activity level (none, moderate, or vigorous), history of chronic diseases (hypertension, diabetes, cancer/malignancy, stroke, coronary heart disease, and kidney dysfunction), serum creatinine and uric acid levels, and dietary sodium and water intake. These variables were included in the regression models, to control for potential confounding factors.

### Statistical analysis

The estimated prevalence of self-reported kidney stones was calculated using NHANES sample weights and survey design variables, to ensure nationally representative estimates of noninstitutionalized population of the United States. Age-standardized prevalence estimates were calculated using the population from the 2020 U.S. census stratified by age group (20–39, 40–59, and ≥60 years).

To examine differences by obesity status over time, we performed subgroup analyses that stratified obesity using a BMI ≥30 kg/m^2^. We applied a survey-weighted multivariate logistic regression model to investigate potential risk factors for kidney stones. To assess trends, we treated each survey period as continuous data that were included in the survey-weighted linear regression models. The *P* value for the trend was calculated to evaluate the significance of the changes over time.

Due to the differences in the prevalence of kidney stones in various demographics, we performed subgroup analyses according to sex, race, and PIR levels. Statistical analyses were conducted using R software, with the significance level set at *P* <0.05.

## Results

### Baseline characteristics of participants

Table 1 summarizes the baseline characteristics of individuals with and without kidney stones from the NHANES 2007–2020 dataset. Kidney stones were more prevalent among older adults, with 41.4% of cases in the group aged 40–59 years and 36.3% in those aged ≥60 years, compared to 22.3% in those younger than 40 years. The sex distribution showed a higher prevalence among males (54.1%) than among females (45.9%). Racial/ethnic disparities were also evident, with non-Hispanic Whites comprising 76.2% of kidney stone cases, significantly higher than Hispanics (11.7%), non-Hispanic Blacks (5.9%), and other racial groups (6.2%). All differences in age, gender, and race/ethnicity between the control and kidney stone groups were statistically significant (*P* < 0.001).

**Table 1:** Baseline characteristics of the population in the NHANES 2007–2020

Variable	Overall (n = 206 372 404)	Control (n = 186 190 353)	Kidney stones (n = 20 182 051)	*P* value
Age, years				
< 40	75 924 408 (36.8)	71 423 803 (38.4)	4 500 605 (22.3)	<0.001
40-59	76 304 564 (37.0)	67 955 202 (36.5)	8 349 363 (41.4)	
≥60	54 143 432 (26.2)	46 811 348 (25.1)	7 332 083 (36.3)	
Gender, n (%)				
Male	99 203 224 (48.1)	88 281 439 (47.4)	10 921 785 (54.1)	<0.001
Female	107 169 180 (51.9)	97 908 914 (52.6)	9 260 266 (45.9)	
Race, n (%)				
Non-Hispanic White	138 327 047 (67.0)	122 950 794 (66.0)	15 376 254 (76.2)	<0.001
Hispanic	28 637 443 (13.9)	26 280 515 (14.1)	2 356 929 (11.7)	
Non-Hispanic Black	22 841 736 (11.1)	21 648 132 (11.6)	1 193 603 (5.9)	
Other race	16 566 178 (8.0)	15 310 912 (8.2)	1 255 265 (6.2)	

### Trends in the prevalence of kidney stones from 2007 to 2020

Table 2 presents the age-standardized prevalence of kidney stones among adults in the United States using the NHANES 2007–2020 data. Overall, the prevalence of kidney stones fluctuated over time, ranging from 8.5% to 11.7%, with a significant increasing trend (*P* for trend = 0.007). Males consistently had a higher prevalence than females; however, only females showed a significant upward trend over time (*P* = 0.010). Non-Hispanic Whites had the highest prevalence (ranging from 9.0% to 13.1%), followed by Hispanics (7.8% to 11.1%) and non-Hispanic Blacks (4.8% to 6.6%). Hispanics exhibited the most significant increasing trend (*P* < 0.001), while Non-Hispanic Blacks showed a modest but statistically significant increase (*P* = 0.017).

**Table 2:** Age-standardized prevalence of kidney stones among U.S. adults by time period in NHANES, 2007–2020

Characteristic	Prevalence, % (95% confidence interval)						*P* for trend
	2007–2008	2009–2010	2011–2012	2013–2014	2015–2016	2017–2020	
Overall	9.4 (7.7–11.1)	9.3 (7.6–11.0)	8.5 (6.9–10.2)	10.4 (8.4–12.5)	11.7 (9.5–14.0)	10.2 (8.4–12.0)	0.007
Male	12.6 (10.0–15.1)	11.0 (8.4–13.6)	8.5 (5.7–11.4)	11.8 (8.8–14.8)	13.5 (10.4–16.7)	11.7 (8.8–14.7)	0.179
Female	6.6 (4.5–8.7)	7.8 (5.8–9.8)	8.7 (5.9–11.4)	9.3 (6.8–11.7)	10.2 (7.3–13.2)	8.9 (6.5–11.4)	0.010
Non-Hispanic White	10.5 (8.1–13.0)	10.4 (8.2–12.7)	9.0 (7.3–10.6)	12.0 (9.0–15.0)	13.1 (9.8–16.3)	10.9 (8.3–13.6)	0.107
Hispanic	7.8 (6.3–9.3)	8.0 (6.0–10.0)	9.8 (7.8–11.8)	9.0 (8.2–9.9)	11.1 (9.5–12.8)	10.3 (6.9–13.6)	< 0.001
Non-Hispanic Black	4.8 (2.6–7.0)	5.2 (3.1–7.3)	5.1 (3.5–6.6)	6.0 (3.8–8.2)	6.6 (4.0–9.2)	6.0 (4.0–8.0)	0.017

### Relationship between obesity and kidney stone risk and trends in the prevalence of obesity over time

The multivariate regression model highlighted several significant predictors of developing kidney stones (Table 3). Females had lower odds of developing kidney stones than males (odds ratio [OR] = 0.714; 95% confidence interval [CI]: 0.610–0.836). Age was a critical factor; individuals aged 40–59 years had an OR of 1.605 (95% CI: 1.360–1.894), and those aged ≥60 years exhibited an OR of 1.595 (95% CI: 1.327–1.917). Racial differences revealed that non-Hispanic Blacks had the lowest risk (OR: 0.433), followed by Hispanics (OR: 0.797) and individuals of other races (OR: 0.744), compared to non-Hispanic Whites. Furthermore, individuals with a history of chronic diseases (hypertension, diabetes, cancer/malignancy, and weak/failing kidneys) and those classified as obese (BMI ≥30 kg/m^2^) had an increased risk of developing kidney stones.

**Table 3:** Multivariable logistic regression model predicting history of kidney stones

Variable	Odd ratios (95% confidence interval)	*P* value
Gender (Ref: male)	0.714 (0.610–0.836)	<0.001
Age (Ref: <40 years)		
40–59 years	1.605 (1.360–1.894)	<0.001
≥60 years	1.595 (1.327–1.917)	<0.001
Race (Ref: Non-Hispanic White)		
Hispanic and Mexican	0.797 (0.691–0.918)	0.002
Non-Hispanic Black	0.433 (0.368–0.511)	<0.001
Other Race	0.744 (0.593–0.933)	0.011
PIR (Ref: ≥2)	1.063 (0.932–1.214)	0.358
Education level (Ref: less than high school)	1.034 (0.890–1.201)	0.660
Marital status (Ref: married/living with partner)	0.834 (0.727–0.956)	0.010
Alcohol consumption (Ref: no)	0.947 (0.817–1.098)	0.468
Smoke (Ref: no)		
Former	1.094 (0.927–1.290)	0.284
Current	1.173 (0.993–1.386)	0.060
Physical activity (Ref: no)		
Moderate	0.936 (0.814–1.076)	0.350
Vigorous	0.892 (0.763–1.043)	0.151
Hypertension (Ref: no)	1.428 (1.202–1.696)	<0.001
Diabetes (Ref: no)	1.494 (1.248–1.788)	<0.001
Coronary heart disease (Ref: no)	0.970 (0.727–1.293)	0.831
Stroke (Ref: no)	0.942 (0.760–1.169)	0.586
Cancer/malignancy (Ref: no)	1.386 (1.149–1.671)	0.001
Weak/failing kidney (Ref: no)	2.010 (1.473–2.744)	<0.001
Serum creatinine	1.000 (0.999–1.001)	0.797
Serum uric acid	1.000 (1.000–1.001)	0.553
Dietary sodium intake (Ref: < 2326.50 mg)		
2326.50–3142.49 mg	0.852 (0.710–1.023)	0.086
3142.50–4150.74 mg	0.865 (0.726–1.031)	0.103
≥4150.75 mg	0.842 (0.708–1.002)	0.052
Water intake (Ref: < 660 g)		
660–1619 g	0.876 (0.758–1.013)	0.073
1620–2999 g	0.988 (0.807–1.209)	0.904
≥3000 g	1.003 (0.861–1.168)	0.969
BMI (Ref: < 30 kg/m^2^)	1.387 (1.227–1.568)	<0.001

BMI, body mass index; PIR, poverty-income ratio.

**Figure 2.**
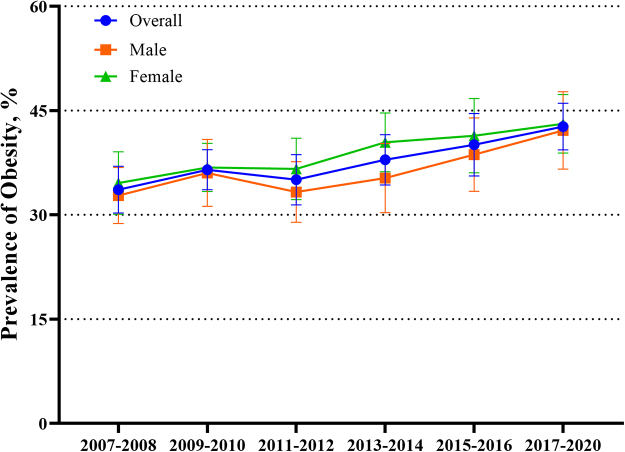
Trends in the age-standardized prevalence of obesity among adults in the United States by sex, from 2007 to 2020.

In parallel, the age-standardized prevalence of obesity showed a consistent upward trend from 2007 to 2020 (Fig. 2). The overall prevalence of obesity fluctuated at approximately 30% in the earlier years but increased to a peak of 42.7% in 2017–2020.

### Sex-based trends in the prevalence of kidney stones among those with obesity

**Figure 3.**
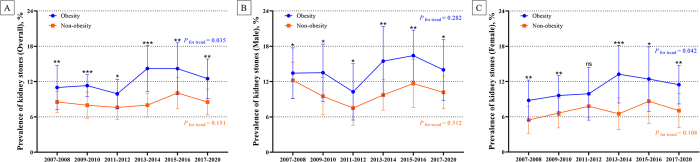
Trends in the age-standardized prevalence of kidney stones among adults in the United States by sex, from 2007 to 2020. (A) Trends in the prevalence of kidney stones by obesity in the overall population. (B) Trends in the prevalence of kidney stones by obesity in males. (C) Trends in the prevalence of kidney stones by obesity in females. *P < 0.05, **P < 0.01, ***P < 0.001, ns: no significant.

To explore the influence of obesity on the prevalence of kidney stones, we analyzed trends stratified by obesity status and sex (Fig. 3A–C and Supplementary Figures S1 and S2, available at: http://links.lww.com/JS9/E398). In the obese population, the prevalence of kidney stones significantly increased from 11.0% (95% CI: 7.2–14.8%) in 2007–2008 to 12.5% (95% CI: 9.1–15.8%) (*P* for trend = 0.035; Supplementary Table 1, available at: http://links.lww.com/JS9/E398). In contrast, the prevalence of kidney stones among nonobese individuals remained stable at approximately 8.5% over the same period (*P* for trend = 0.151).

**Figure 4.**
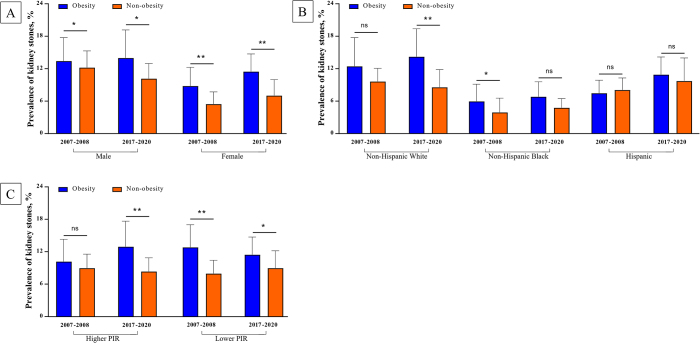
Comparison of the age-standardized prevalence of kidney stones in 2007–2008 vs. 2017–2020 by obesity. (A) Prevalence of kidney stones in 2007–2008 vs. 2017–2020 by obesity and sex. (B) Prevalence of kidney stones in 2007–2008 vs. 2017–2020 by obesity and race. (C) Prevalence of kidney stones in 2007–2008 vs. 2017–2020 by obesity and PIR. *P < 0.05, **P < 0.01, ns: no significant.

Among males, the prevalence of kidney stones among individuals with obesity showed a slight increase from 13.4% to 14.0% from 2007 to 2020, whereas males without obesity experienced a moderate decrease from 12.2% to 10.2% (Fig. 4A). Among females, both the obese and nonobese groups exhibited moderate increases in the prevalence over time. Notably, the prevalence among females with obesity increased significantly from 8.8% to 11.5% (*P* for trend = 0.042), whereas that among females without obesity exhibited a less pronounced increase from 5.4% to 7.0% (*P* for trend = 0.108).

### Race-based trends in the prevalence of kidney stones prevalence among those with obesity

**Figure 5.**
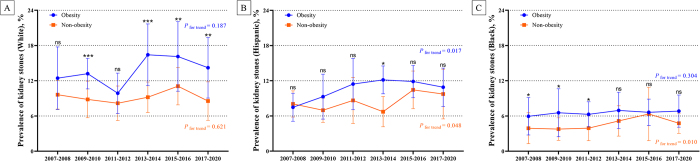
Trends in the age-standardized prevalence of kidney stones among adults in the United States by race, from 2007 to 2020. (A) Trends in the prevalence of kidney stones by obesity in non-Hispanic Whites. (B) Trends in the prevalence of kidney stones by obesity in Hispanics. (C) Trends in the prevalence of kidney stones by obesity in non-Hispanic Blacks. *P < 0.05, **P < 0.01, ***P < 0.001, ns: no significant.

Trends in the age-standardized prevalence of kidney stones among U.S. adults with obesity by race from 2007 to 2020 are shown in Figures 5A–C and Supplementary Figures S3 and S4, available at: http://links.lww.com/JS9/E398. Among non-Hispanic Whites with obesity, the prevalence modestly increased from 12.4% (95% CI: 7.1–17.8%) in 2007–2008 to 14.2% (95% CI: 9.1–19.4%) in 2017–2020 (*P* for trend = 0.187; Supplementary Table 1, and Figure 4B, available at: http://links.lww.com/JS9/E398). The prevalence among individuals without obesity in this group remained stable at approximately 9% across the years (*P* for trend = 0.621).

Among Hispanics with obesity, the prevalence increased from 7.5% (95% CI: 5.1–9.9%) in 2007–2008 to a peak of 12.1% (95% CI: 9.8–14.5%) in 2013–2014, followed by a slight decrease to 10.9% (95% CI: 7.6–14.2%) in 2017–2020 (*P* for trend = 0.017). Hispanic individuals without obesity also exhibited a significant increase, from 8.0% to 9.7% (*P* for trend = 0.048).

Among non-Hispanic Blacks with obesity, the prevalence remained relatively stable, increasing slightly from 5.9% in 2007–2008 to 6.8% in 2017–2020 (*P* for trend = 0.304). However, non-Hispanic Blacks without obesity showed a significant increase from 3.9% to 4.8% (*P* for trend = 0.010).

### PIR-based trends in the prevalence of kidney stones among those with obesity

**Figure 6.**
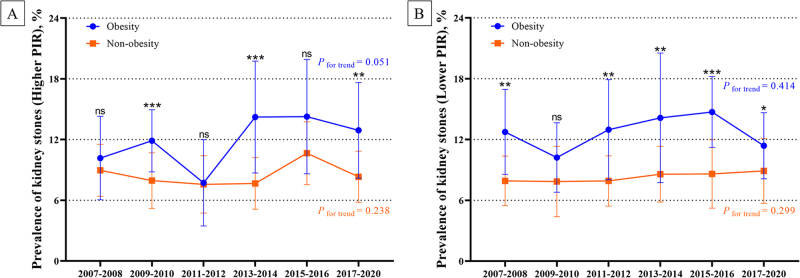
Trends in the age-standardized prevalence of kidney stones among adults in the United States by PIR, from 2007 to 2020. (A) Trends in the prevalence of kidney stones by obesity in a high PIR. (B) Trends in the prevalence of kidney stones by obesity in a low PIR. *P < 0.05, **P < 0.01, ***P < 0.001, ns: no significant.

Figures 4C and 6 illustrate the trends in the age-standardized prevalence of kidney stones according to the PIR and obesity status between2007 and 2020. Among individuals with obesity and a higher PIR (≥2), the prevalence of kidney stones fluctuated over time, starting at 10.2% (95% CI: 6.0–14.3%) in 2007–2008, peaking at 14.3% (95% CI: 8.6–19.9%) in 2015–2016, then declining slightly to 12.9% (95% CI: 8.2–17.6%) in 2017–2020 (*P* for trend = 0.051; Supplementary Table 1, available at: http://links.lww.com/JS9/E398). Among individuals with without obesity and a high PIR, the prevalence remained low and stable, ranging from 9.0% to 8.3% (*P* for trend = 0.238).

Individuals with obesity and a low PIR (<2), had a notably high prevalence of, beginning at 12.8% in 2007–2008, with slight fluctuations, and ultimately decreasing to 11.4% in 2017–2020 (*P* for trend = 0.414). Individuals without obesity and with a lower PIR showed a lower and more stable prevalence, ranging from 7.9% to 8.9% over the study period (*P* for trend = 0.299).

## Discussion

Our analysis of NHANES data from 2007 to 2020 revealed a significant upward trend in the prevalence of kidney stones among adults in the United States, particularly among individuals with obesity. The age-standardized prevalence of kidney stones among individuals with obesity increased from 11.0% to 12.5%, underscoring the growing obesity-associated health burden. Furthermore, our study identified significant demographic disparities in the prevalence of kidney stones among adults with obesity, with notable variations according to sex, race/ethnicity, and socioeconomic status. These findings provide critical insights for addressing the inequities in the risk of kidney stones and tailoring interventions for vulnerable populations. To the best of our knowledge, this study is the first to systematically evaluate contemporary trends in the prevalence of kidney stones in individuals with and without obesity, thereby offering a comprehensive understanding of the influence of obesity on kidney stone formation across diverse demographic groups.

The overall increase in the prevalence of kidney stones in our study is consistent with previous research at the national level. Notably, previous NHANES analyses documented an increase in prevalence from 5.2% in 1988–1994 to 8.8% in 2007–2010^[1,10]^, which is consistent with our findings of continued upward trends. In contrast, the Global Burden of Disease (GBD) 2021 study reported a notable increase in the incidence of urolithiasis, along with a decreased global age-standardized rate, highlighting the critical role of demographic changes such as population growth and aging, as well as the importance of standardizing data to a reference population when interpreting epidemiological trends^[12]^. Moreover, these global aggregates mask significant regional and demographic variabilities. For example, recent projections using GBD 2021 data indicate that while the incidence and mortality rates of urolithiasis are expected to decline or stabilize in China and India, the United States is projected to experience a steady and more pronounced increase in the incidence and mortality through 2050^[13]^. Methodological considerations, such as differences in data sources, case definitions, and diagnostic practices, contribute to the variability in reported trends across studies. Our finding of an increasing prevalence of kidney stones in the United States aligns with these nuanced national trends and underscores the importance of localized epidemiological analysis. Thus, this evidence emphasizes that while global trends provide a valuable context, we should consider critical regional and population-specific patterns that are essential for guiding targeted prevention and public health strategies.

The upward trend in kidney stone prevalence among adults with obesity in the United States aligns with the growing obesity epidemic^[14]^, and obesity has been established as a major risk factor for kidney stones^[15]^. The significant rise in increase in the prevalence of kidney stones among individuals with obesity likely reflects multifactorial pathways. Obesity-induced metabolic disturbances promote stone formation via insulin resistance, which decreases renal calcium reabsorption, and chronic low-grade inflammation, which affects urinary citrate excretion.^[16-19]^ Diet also plays a role; high sodium intake, which increases urinary calcium levels, and elevated animal protein consumption, which increases uric acid levels, commonly observed in obese populations and exacerbate the risk of developing kidney stones^[20,21]^. Furthermore, inadequate hydration – due to altered thirst perception^[22]^ and preference for dehydrating beverages such as sugar-sweetened sodas – reduces urine volume and increases the urinary supersaturation of stone-forming salts^[23]^. Physical inactivity, which is prevalent among individuals with obesity, further diminishes urine flow and facilitates crystal aggregation^[24,25]^. These insights emphasize the urgency of targeted interventions addressing obesity-related metabolic and lifestyle risk factors, to reduce the burden of kidney stones.

Our study highlighted significant sex disparities in the prevalence of kidney stones, with higher rates in males but a narrowing sex gap driven by distinct trends. The sharp increase in the prevalence of obesity among females during the study period likely contributed to the increasing kidney stone burden in females with obesity^[5]^. Female-specific factors, such as physical inactivity, pregnancy history, hormone therapy, and menopause, also influence the risk of kidney stones by modifying the urinary composition^[25-27]^. Among individuals without obesity, the trends also revealed important sex differences. Males without obesity experienced a moderate decrease in the prevalence of kidney stones, whereas females without obesity showed an upward trend. This divergence may partly reflect the increasing prevalence of lean diabetes among females, a metabolic condition associated with a risk of developing kidney stones, even in the absence of obesity^[28]^. These findings suggest that obesity management programs should integrate kidney stone prevention strategies, such as hydration education and dietary counseling, particularly for females with obesity. Primary care providers should consider routine urine screening for females with obesity and metabolic syndrome.

Racial and ethnic disparities in the prevalence of kidney stones vary according to the obesity status. Non-Hispanic Whites consistently had the highest prevalence of kidney stones, with a modest increase in the prevalence among individuals with obesity. This trend parallels a significant increase in the prevalence of obesity among non-Hispanic Whites^[6]^. The strong association between obesity and the prevalence of kidney stones in non-Hispanic Whites may be caused by genetic predispositions that contribute to both conditions^[29]^. Among Hispanics, the prevalence increased significantly among individuals with and without obesity, suggesting that factors beyond obesity, such as genetic predisposition, socioeconomic challenges, or comorbid conditions such as gout, contribute to the risk. Previous studies have documented a rising prevalence of gout among Hispanic adults, which may exacerbate the risk of developing kidney stones^[30]^. In contrast, the prevalence among non-Hispanic Blacks with obesity remained stable, although non-Hispanic Blacks without obesity showed a significant increase. These findings indicate that community-based interventions should specifically target Hispanic and nonobese non-Hispanic Black populations, focusing on kidney stone and comorbid condition management.

In addition to biological factors, socioeconomic determinants can modulate the risk of kidney stones in complex ways, as reflected by the shifting trends in the PIR. Historically, a lower PIR, which often correlates with a lower socioeconomic status, has been associated with a higher prevalence of kidney stones^[31]^. For example, in 2007–2008, individuals with obesity in the lower PIR group had a higher prevalence of kidney stones than those in the higher PIR group. This disparity might be explained by socioeconomic factors such as limited access to healthcare, poorer dietary habits, and higher stress levels, which collectively heighten the risk of kidney stones among individuals with obesity and lower incomes ^[32-34]^. By 2017–2020, this trend shifted; individuals with obesity in the higher PIR group showed a greater prevalence of kidney stones than those in the lower PIR group. This reversal might reflect two concurrent phenomena. First, the expanded healthcare coverage following the Affordable Care Act likely enhances the management of obesity-related metabolic conditions among individuals with a lower PIR^[35]^. Second, individuals with higher a socioeconomic status, despite having better access to healthcare and resources, may be increasingly exposed to lifestyle-related risk factors, such as high-calorie diets or sedentary behaviors, which contribute to both obesity and kidney stone formation^[23,25,36]^. These findings highlight the necessity for tailored public health strategies that improve healthcare accessibility and lifestyle support for lower-income populations and simultaneously implement targeted prevention and education initiatives aimed at mitigating lifestyle risks among higher-income groups.

This study benefits from the use of nationally representative NHANES data, which allow for generalizability to the broader population of the United States. The inclusion of multiple survey cycles enabled a robust assessment of trends over time and provided valuable insights into the evolving burden of kidney stones. However, this study had several limitations. Primarily, the reliance on self-reported histories of kidney stones may have introduced recall bias, and the lack of imaging or laboratory confirmation may have affected the accuracy of the prevalence data. Therefore, we advocate multi-institutional collaborations to develop and deploy cost-effective screening protocols, such as ultrasound-based screenings, in future epidemiological studies. Additionally, our multivariate logistic regression model did not incorporate important confounding factors, such as continuous, objective hydration measurements and the family history of kidney stones, owing to data limitations within the NHANES. Finally, the cross-sectional design limited the causal inference between risk factors and kidney stone formation. Prospective longitudinal studies are warranted, to elucidate the underlying mechanisms driving these trends and evaluate the efficacy of targeted prevention and intervention strategies.

## Conclusions

This study highlights the increasing prevalence of kidney stones in the United States, particularly among individuals with obesity. Our findings emphasize the urgent need for comprehensive strategies aimed at addressing obesity and reducing the disparities in the prevalence of kidney stones, particularly among females and Hispanics. Future studies should explore longitudinal associations and investigate the effectiveness of targeted interventions to address this growing public health challenge.

## Data Availability

The datasets generated during and/or analyzed during the current study are publicly available. The data can be found here: the National Health and Nutrition Examination Survey dataset (https://www.cdc.gov/nchs/nhanes/index.htm).
